# The Rheometric Analysis of the Polymer Modifier’s Properties in the Environment of Hydrated Cement

**DOI:** 10.3390/ma14051064

**Published:** 2021-02-24

**Authors:** Khrystyna Moskalova, Aleksej Aniskin, Goran Kozina, Božo Soldo

**Affiliations:** 1 Department of Processes and Apparatuses in the Technology of Building Materials, Odessa State Academy of Civil Engineering and Architecture, 65029 Odessa, Ukraine; 2 Department of Civil Engineering, University North, 42000 Varaždin, Croatia; aaniskin@unin.hr (A.A.); gokozina@unin.hr (G.K.); bsoldo@unin.hr (B.S.)

**Keywords:** rheometry, polymers, methylhydroxyethylcellulose, ethylene vinyl acetate, sodium salt, cement structure

## Abstract

This paper investigates the effect of polymer modifiers (re-dispersible powder, multifunctional additives, methylhydroxyethylcellulose) on the rheological behavior of emulsions, saturated of calcium hydrosilicates to simulate a hydrating cement structure. The subjects of the study were modified emulsions which had varied concentrations of each additive and they were examined comparatively to a base emulsion. Tests were performed with a CR-rheometer (“Himpribor-1”, Tula, Russia) applying the Searle measuring principle at various shear rates to characterize viscosity properties. The performance of modified mixtures within the operating period was analyzed by using two parameters—effective viscosity (η) and the proportion of structural failure (|m|). The test results showed that the most important factor influencing rheological characteristics is the addition of methylhydroxyethylcellulose additive—the higher additive amount in the emulsion, the higher the viscosity. Furthermore it was noted in the work that adding olefin sulfonate sodium salt causes reduced viscosities as well as lower shear moduli. If ethylhydroxyethylcellulose and ethylene vinyl acetate additives are used in the same mixture together, the rate of structural failure |m| can be relatively similar and low regardless of whether the mixture has large or small viscosity values.

## 1. Introduction

Rheological characteristics of modified building mixtures exert considerable effects on the physical–mechanical and technological reaction of the final materials. Building mortars from dry building mixtures (DBM) is a dynamically developing branch of the building materials industry [1,2]. Dry mix mortar comes in a broad range of products, among others dry mix products as well as additives in dry condition. During the last 50 years, a large number of materials for the production of dry building mixtures have been investigated, and production techniques have been developed and used. Currently, the following are used for the development of special plaster mixes: water-retaining additives, plasticizers, water repellents, additives that improve adhesive properties, etc. [3,4]. The most common polymer modifiers for DBM are re-dispersible polymer powders (RP) [5,6,7,8], which are prepared by emulsion polymerization or copolymerization of polymers in the dispersion state; after further spraying and drying methods, they are able to create a dispersion in water (re-dispersible) [9]. After mixing the RP with water it is instantly and completely re-dispersed, and disperse particles combine and form films with tensile strength for nearly 4.9 MPa. These polymer films are in the cavities, microvoids and in the pores of the hardened mixtures, practically reinforcing them, which leads to the increase in bending strength and tearing from the base-adhesion. Hence, they can be used to increase the homogeneity of the hardened mortar, increase the bond strength, compressive and tensile strength under bending, deformation property, water absorption, frost resistance, etc. [3,5,7]. Cellulose ethers are effective additives to increase the water-holding properties of dry building mixtures [10]. If in a thin layer an ordinary cement–sand mixture can provide a water retention of 75–80%, then a water-retaining additive increases this figure to 97–98% [4,11]. Dissolved cellulose molecules form aqua complexes that hold water firmly by intermolecular interaction. Moreover, the addition of cellulose leads to the significant increase in viscosity and prevents the sedimentation of cement and filler particles [12]. Some authors [13,14] have noticed that adding cellulose leads to an increase in viscosity of the mortar solutions, and it can raise or lower the yield stress and viscosity of the mortars depending on their content. In the work [15] such reduction in base of the steric hindrance caused by the cellulose adsorbed onto cement particles was described. Similarly, Brumaud [16] noted that the yield stress of cement pastes increases as a result of bridging flocculation caused by the adsorbed admixture. In the work [17] it was discovered that the extent to which individual admixtures have an impact on mortar rheology is closely related to a particular kind of cellulose admixture. The most noticeable changes in consistency were observed for hydroxypropylmethyl cellulose and hydroxyethylmethyl cellulose-based admixtures, in which cases the viscosity increased, from approximately 6000 MPa∙s to approximately 30,000 MPa∙s; when the viscosity of cellulose admixtures was even more increased, it had a lesser impact on the mortar consistency. The authors in [18] think that variations given in the literature stem from the diverse structure and molecular weight of the cellulose polymers used. Therefore, the nature of the additives and further admixtures in the mix influence the mechanism of action of these admixtures and their effect on the rheological properties. For instance, Patural [15] worked with cellulose with lower molecular weight than Brumaud [16]. Compared to previous investigated suspensions, our studies have concerned cellulose with larger particle size varying to 125 μm (cellulose methyl ether). 

A common element amongst all of the abovementioned additives is that they transform binder properties, i.e., viscosity and surface tension. As a consequence of building mixtures alteration, binder behavior may turn out more complex and more difficult to characterize. This means that traditional properties of building mixtures such as workability, consistency, and common rheological parameters could be insufficient in the process of describing the behavior of modified building mixes, because such methods are intrinsically devised to be used with unmodified building materials [19]. Additionally, mixtures for mechanized application should have the technological properties of hand-applied mixtures, and after a brief mixing with water should provide uniformity, have good pumpability and maintain the working properties for a given time [20]. Furthermore, not many studies can be found on the rheology of cementitious systems comprising altogether ethylene vinyl acetate copolymer, water retention agents and air entraining additive; most of the studies deal with the only one admixture. Nevertheless, for the purposes of the majority of these studies, a single dosage of the additives was used, or they were carried out at a constant shear rate [21,22,23,24,25]. Therefore, the goal of this research is to analyze the impacts of dosages of three common additives on mixtures’ rheology and to reach a deeper understanding of the flow properties of multicomponent building mixtures that can help scientific justification of the formation processes in cement–polymer compositions.

## 2. Materials and Methods 

### 2.1. Materials

In this study, an emulsion with three different additives was used as a testing mixture. To preparing testing emulsions we use aqueous solutions of calcium hydroxide, which are called limewater. They were obtained by mixing lime and water: CaO + H_2_O → Ca(OH)_2_. Later, chemical additives are added to limewater for obtaining emulsion. Limewater simulates the environment of a hydrated cement due to formation of calcium carbonate. The concentration of additives was selected based on the recommendations of the supplement manufacturer. All additives with their selected physical and chemical properties are given in Table 1.

The first was a re-dispersible powder additive Vinnapas 5034N (Wacker Chemie AG, Munchen, Germany), as it is referred to on the market. This additive is a copolymer powder of vinyl acetate and ethylene. Vinnapas 5034N can increase bond strength and strength due to the formation of the so-called resin domains or polymer films [9]. The concentrations of this additive were: 3.5 w.p., 8 w.p. and 12.5 w.p. from 100 weights part of emulsion. The second one was an air entraining additive, wetting and plasticizing agent known commercially as Hostapur OSB (Clariant AG, Basel, Switzerland) [26]. This additive is an olefin sulphonate, sodium salt. Hostapur provides quick wetting and dispersion of building mixtures (e.g., of machine applied plasters and renderings), reduces stickiness and improves workability and pumpability of wet mortars. Three dosages of this particular additive were analyzed for the purpose of the present study 0.05 w.p., 0.15 w.p. and 0.25 w.p. from 100 weights part of emulsion, and the last one was water-retentive additive Tylose MH60010 P4 (Shin-Etsu Chemical, Tokio, Japan) [27], a cellulose methyl ether, 2-hydroxyethyl ether, in dosages 0.8 w.p., 1.25 w.p. and 2 w.p. from 100 weights part of emulsion.

Additives were blended with a base binder for 5 min to achieve homogeneous state and then placed in a working tool for testing. The minimum concentration of all polymers in suspensions amounts to 4.17% by weight in a solution of Ca(OH)_2_ and such mixture is called “E”; 12.85% is then the maximal concentration of three polymers (indicated as “VHT” mixture). The present paper aims to demonstrate some of the particular qualities observed in rheological tests of 15 water emulsions with three different polymer additives. To achieve the aims of the study, the experiment was carried out according to the Box–Behnken design. They introduced three level designs for fitting response surfaces. The 2 k factorials are combined with incomplete block designs to form such designs. The resulting designs generally prove to be quite efficient when it comes to the number of required runs [28].

### 2.2. Testing Method

**Figure 1:**
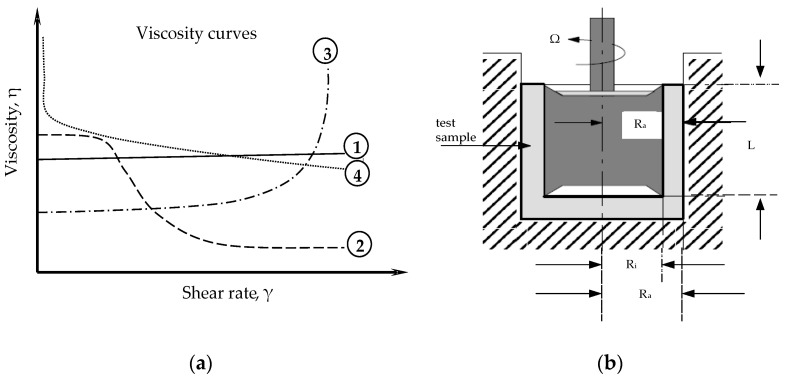
(**a**) Different types of fluid flow: Newtonian fluid (1), Pseudoplastic fluid (2), Dilatant fluid (3), Pseudoplastic fluid with yield strength (plastic fluid) (4); (**b**) Model of a CR-rheometer with a Searle-type measuring system combined with coaxial cylinder, where Ω—angular velocity [rad/s], R_a_—radius of the cup [m], R_i_—radius of the rotor [m], L—rotor height [m].

Modified cement mortars are complicated dispersion systems and they show specifications of non-Newtonian pseudoplastic fluids during the flow; their viscosity depends on shear stress. At a low shear rate, the effect of shear orientation is small, and all molecules or particles in the liquid make a random Brownian motion. At very low shear rates, pseudoplastic fluids, show the same behavior as Newtonian fluids with a certain viscosity η_0_, which is not affected by shear rate. When the shear rate increases and reaches a certain value (ultimate shear stress τ_0_), the viscosity of the liquid drops sharply and it begins to flow [29,30]. The manifestation of thixotropic behavior is characteristic of pseudoplastic fluids (Figure 1a), due to the fact that no matter how quickly the particles disorient, they quickly orient themselves again. The flow character of such systems can be described using the rotational method of viscometry. The principle of operation of a viscometer is based on measuring the moment of shear resistance of the test material placed in the gap between the sensing elements when one of them (internal) rotates at a constant angular velocity, by converting the twist slope of the elastic element into a time interval which is proportional to viscosity. The scheme of testing procedure is illustrated in Figure 1b. 

The rotational rheometer used in this study were “Polimer” RPE-1M (manufacturer “Himpribor-1”, Tula, Russia) with coaxial cylinders measuring system according to DIN 53018. The inner cylinder, i.e., the rotor, is run by a motor. Its speed is monitored for constant or programmed rotor speeds. At the same time, the second cylinder, i.e., the cup, maintains at rest. The test was run from the lowest speed gradient 0.066 to the highest 134.5 s^−1^ and back. To eliminate errors, the viscosity of each type of emulsion was measured sequentially 3 times by turning the rotor on and off. The average value of these measurements was taken as the measurement result. The duration of the study for each sample was 8 h. The tests cup is jacketed at the thermostatic chamber in order to maintain a constant material temperature 19 ± 1 °C. The liquid in the annular gap is propelled to flow by the driven inner cylinder. It is possible to get minimized errors during testing by making the gap size smaller between cylinder, linearizing the velocity gradient across R_a_-R_i_ (Figure 1b) [30]. So, we used type T2-B30 cylinders. The inner diameter of the outer cylinder is 24 mm. The outer diameter of the inner cylinder is 19.863 mm. To define the annular gap size, we used the ratio of the radii, δ = 0.83. In accordance with the DIN 53018 standard it allows the use of devices in which, lies in the range: 1 < δ < 1.10. The annular gap is of constant width: the test may be run with samples that contain particles with a particle size less than 1/3 of the gap size. The outcome of the resistance of the liquid being sheared between the stationary and rotating boundaries of the sensor system is a viscosity-related torque operating on the inner cylinder, counteracting the torque produced by the drive motor. Due to the torque applied a spring twist, this torque detector is put between the drive motor and the shaft of the inner cylinder. The twist slope of the torque spring then serves as a direct measure of the viscosity of the sample. 

For the mathematical description of the rheological behavior of non-Newtonian fluids, the models by Newton, Ferry, Steiner, Ostwald-de-Waele, Bingham [30] and others are used, which describe the rheological behavior of liquids quite well. The commonly-used model is the Ostwald-de-Waele Equation (1) that requires two parameters, i.e., effective viscosity and rate of structural failure. Flow curves and viscosities constructed in double logarithmic coordinates are straight lines. After Logarithm (2) the Ostwald-de-Waele equation becomes linear in parameters:

η = K ∙ (γ′)^m^, (1)

lnη = lnK + m ∙ lnγ′,(2)

The coefficient K equals the effective viscosity η, Pa∙s, at shear rate γ′ = 1 s^−1^, and the rate of structural failure during shear deformations is characterized by the exponent m < 0—the higher |m|, the less stable is the fluid structure during flow (defined as the slope of the line) [30]. 

## 3. Results

### 3.1. Rheology of Modified Emulsions

**Figure 2:**
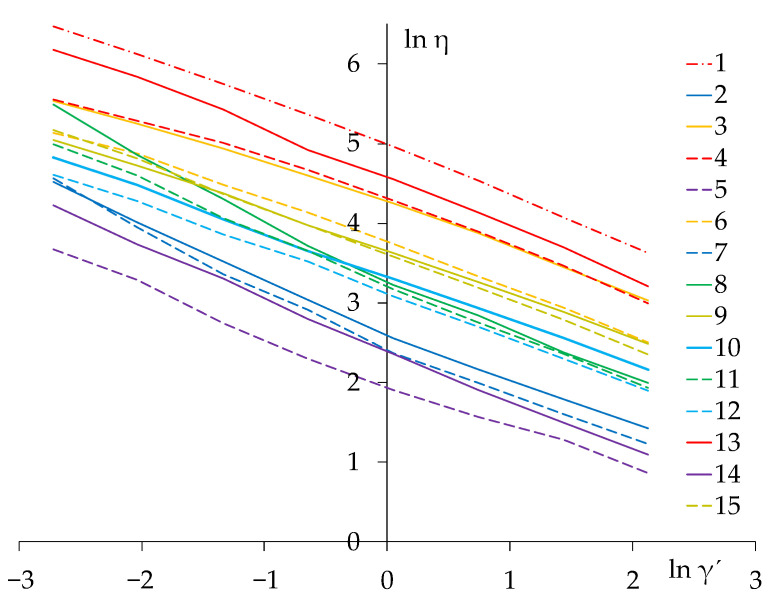
Viscosity curves.

The data obtained during the experiment on the change in the effective viscosity η (Pa∙s) of compositions with different polymer matrices, the compositions of which changed according to the three-factor plan (Table 2), made it possible to estimate the parameters of the Ostwald-de-Waele function Table 3. Models in the Table 3 and Table 4 are constructed with average experimental error S_a_{ln K} = 0.021 and S_a_{m} = 0.012. Zone inadequacy error γ′ = 1 does not exceed 5% as can see in Table 3. Which, on the basis of rheological parameters, can be considered satisfactory for engineering conclusions [30]. The effects of the applied additives on the viscosity are presented in Figure 2. Compositions No. 1, 13, 3, 4, 6 demonstrate the highest 5 viscosities, achieved by maximum amounts of Tylose. As expected, the lowest viscosity is in compositions with minimum amounts of the water-retaining additive, 4 lines can be seen on the bottom of Figure 2. When the polymer molecule of cellulose ether is dissolved in the liquid phase of the hardening system, the viscosity of the aqueous phase increases significantly. The molecules of dissolved cellulose form aqua complexes, firmly holding water by intermolecular interaction forces. Unlike with other additives, there is a greater reaction of cellulose with water due to the preponderance of hydroxyl functional groups. The water molecules interact promptly with the cellulose chains and form hydrogen bonds immediately [31]. 

On the one hand, the cellulose methyl ether additive increased viscosity and this impact is in direct relation to the additive dosage. Nevertheless, we can see compositions No. 9, 10, 12 where after combined dosage of Vinnapas and medium dosage of Tylose the viscosity and |m| are reduced. The degree of destruction of the structure |m| indicates the different effects of additives on the ability of the mixture to deform in the process of its technological processing and application. 

Low values of the rate of structural failure are also characteristic for emulsions with high viscosity. So, emulsions No. 3, 4 show |m| = 0.52 at a viscosity of η ≈ 70 Pa∙s, while emulsion No. 9 also has a low |m| = 0.53 and at the same time viscosity η = 37.7 Pa∙s. It is important to note that No. 9 contains a maximum of vinylacetate Vinnapas and an average dosage of hydrophobic agent (sodium salt) Hostapur and cellulose Tylose. Polymers have segments distributed along the chain that tend to interact with each other. Consequently, intramolecular and intermolecular associations between the polymer chains may be formed, so that a three dimensional network is created and the viscosity of the interstitial solution is increased. Adding salt facilitated intramolecular associations in the solutions and the increase of the viscosity was delayed. In [32], the authors have noted that in more concentrated solutions, the coil contraction after adding salt resulted in lower viscosities and lower shear moduli. Therefore, we can conclude that selecting a particular hydrophobic group is of great importance for the control of the drift in composition. These figures show that it is possible to obtain mixtures with a low viscosity—this facilitates ease mixing of the mixtures and a stable structure during application. 

The viscosity also increases as the Tylose ratio increases in the mixtures with minimum and a medium amount of Vinnapas as seen in compositions No. 6, 10, 12, and the rate of structural failure during shear deformations can be relatively low |m| ≈ 0.55. In composition No. 6 and No. 10 the amount of Tylose increased from 1.25 w.p. to 2 w.p. with a minimum 3.5. w.p. dosage of Vinnapas, and, as we can see, this change increases viscosity from 27.25 Pa∙s to 42.14 Pa∙s, but the index |m| is stabilized on the 0.55. 

Emulsion No. 10 and No. 12 contain a medium 1.25 w.p. dosage of Tylose, in this case, viscosity decreases by 27% from 27.25 Pa∙s to 21.89 Pa∙s when the amount of Vinnapas increases from 3.5 w.p. to 8 w.p., at the same time |m| increases just by 1.8%. 

Accordingly, it can be concluded that Tylose is more effective regarding mixture stabilization in the emulsion where both Vinnapas and Tylose are added. At this step of the study it is hard to describe which additives have the highest effects on the structure. 

### 3.2. Effect of Additives on Destruction of the Structure 

**Figure 3:**
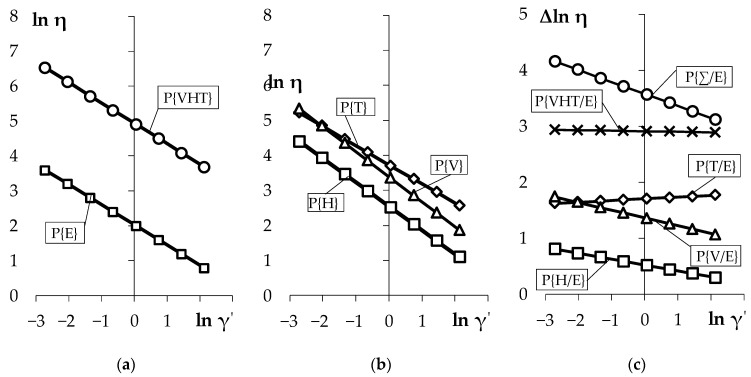
Logarithmic functions of the viscosity: (**a**,**b**) emulsions P; (**c**) increments of the logarithm of viscosity in relation to the reference emulsion P{E}.

Further information for all emulsions was obtained using a special technique for the analysis of logarithmic viscosity functions (Table 3). This methodology describing the effects of interaction of polymer modifiers was initiated in [33]. Five functions are used for comparative analysis of rheological behavior of emulsions: with a minimum (4.17%) and maximum concentration (12.85% by weight) of three components (reference P{E} indicated in Table 2 No. 5 and P{VHT} indicated in table like No. 1) and with an extremely high content of one of the components (P{V}, P{H} and P{T}). The results are presented in Figure 3. The first Figure 3a shows a logarithmic line of viscosity with minimum P {E} and maximum P{VHT} concentration of polymer modificators. The effective viscosity decreases almost 16 times with an increase in shear rate from 39.4 Pa∙s to 2.4 Pa∙s in the emulsion with minimum amount of additives. Viscosity decreases from 644.6 to 37.5 Pa∙s—a drop of 17 times in the emulsion with maximum concentration of all three chemical additives. The reasons for the increase viscosity in the mixture P{VHT} compared to P{E} are due, firstly, to an increase in the concentration of polymer additives by 2.5–5 times by mass as well as a reduction in the specific volume Ca(OH)_2_; secondly, to an increase in the ratio of physically bound water (in the process of dispersing, swelling and dissolving polymer additives).

The second Figure 3b demonstrates the viscosity parameter results with an increased concentration of single emulsion component (Vinnapas, Hostapur or Tylose). Figure 3b shows that three of the investigated emulsions are within the viscosity-limited region of the emulsions with minimum and maximum amount of additives, and form ranked range η (Pa∙s) in γ′ = 1 s^−1^, P{VHT} = 142.6 > P{T} = 42.14 > P{V} = 25.2 > P{H} = 10.69 > P{E} = 6.71. 

A methodology has been developed for a comparative analysis of viscosities changing under the influence of changing prescription factors (x_1_, x_2_, x_3_) using the differences of the logarithmic functions described by Ostwald-De-Waele models (3). One of the functions is for the mixture “E”, considered as a reference. For other mixtures “U”, direct-reflected functions (3) of the logarithm differences are determined:

exp([lnK_U_ − *m*_U_lnγ′] − [lnK_E_ − *m*_E_lnγ’]) = exp(lnη_U_ − lnη_E_) = η_U_/η_E_, (3)

Viscosity changes in relation to the reference emulsion are reflected in the graphs in Figure 3c. The effect of the separate usage of additives on viscosity is given in Table 4.

It is seen that the introduction of a water-retaining additive has the greatest effect on viscosity P{T}/P{E} = 5.5. Moreover, such an effect is not dependent on γ’ (the graph is parallel to the abscise axis), and the rate of structural failure during shear deformations differs little from zero |m| = 0.03. These results also confirm the previous finding of increased viscosity in emulsions with different amounts of additives. So, since the cellulose polymer cannot be dissolved in water, it has a compact and ordered structure, and it is very stable physically and chemically. After cellulose chains and water molecules interact, a lot of intermolecular hydrogen bonds are cut from the cellulose/water interface. However, these chains form new connections with the water molecules [34]. This behavior of Tylose lead to an increase of the ratio of physically bound water and consequently viscosity while keeping the mosaic shape of the structure and its destruction during shear deformations. In addition, due to steric interactions of the cellulose chains, the CaOH_2_ grains and aggregates of other additives move apart. However, the longer the cellulose chains are, the greater their interaction becomes, leading then to a more stable suspension. The sensitivity of effective viscosity η to the amount of re-dispersible powder additive Vinnapas is somewhat lower. The parameter in question changes by 3.9 times; viscosity increases because of the strong films formed during dispersion. The viscosity of the mixture P{H}/P{E} = 1.7 is somewhat increased with Hostapur, its plasticizing effect is not explicitly detected.

In the study, we expected to see additivity from the introduction of all three polymer modifiers. (3e) shows the calculated amount of viscosity increase when all three polymers are simultaneously added to the emulsion in the maximum amount. However, as we can see, the empirical growth function P{VHT}/P{E} = 18.4 and it is 2 times lower than expected. The hypothesis of additivity is not confirmed. This phenomenon cannot be controlled and depends mainly on the way in which the additives molecules react in a mixture with other additives and compounds. This was confirmed by high variability indices of the examined properties.

## 4. Conclusions

This study primarily aimed to evaluate and explain the impact of three representative chemical additives on emulsion rheology and performance. The rheological characteristics were examined at varied speed gradients using the CR-rheometer applying the Searle measuring principle. On the basis of the study results, the following can be concluded:

- In polymer emulsions, the greatest influence on viscosity is exerted by a water-retaining additive Tylose P{T}/P{E} = 5.5, indicating a fairly “strong” frame created by cellulose ethers and this effect is greatly dependent on the amount of the cellulose additive. Cellulose reacts a lot more with water due to the preponderance of hydroxyl functional groups. Due to steric interactions of the cellulose chains, the CaOH_2_ grains and aggregates of another polymer additives move apart. However, the longer the cellulose chains are, the greater their interaction becomes, leading then to a more stable emulsion.
- For emulsions with high viscosity, high values of the rate of structural failure are also characteristic. However, the rate of |m| can be relatively low. Emulsion No. 9, with maximum dosage of vinylacetate Vinnapas (12.5 w.p.), an average dosage of hydrophobic agent (sodium salt) Hostapur (0.15 w.p.) and cellulose Tylose (1.25 w.p.), demonstrates a good stabilized structure with |m| = 0.53 at a viscosity of η = 37.7 Pa∙s. Polymers can create a stable and strong connection with each other due to theirs segments distributed along the chain that tend to interact with each other.
- The viscosity of emulsions increases about 18 times when switching from a minimum to a maximum content of polymer components. Presumably the reference emulsion contains a mosaic structure with weak intermolecular bonds, which increase with increasing concentration of polymers. However, the additivity hypothesis from the simultaneous introduction of all three polymer modifiers in the maximum amount in an emulsion is not confirmed.
- The plasticizing effect of Hostapur is not explicitly detected. The chemical structure of this polymer additive does not significantly effect the rheological behavior of the whole investigated system. However, we have noticed that adding sodium salt enhanced intramolecular association in the solutions while delaying the increase of the viscosity.

## Figures and Tables

**Table 1:** Additives used in the study and their physical and chemical properties.

Property	Vinnapas 5034N	Hostapur OSB	Tylose MH60010 P4
physical condition and color	white to light beige powder	white–slightly beige dry powder	white powder
contain of active substance	min. 98% copolymer powder of vinyl acetate and ethylene	90–98% olefin sulphonate, sodium salt	90–95% cellulose methyl ether, 2-hydroxyethyl ether
bulk density	400–550 kg/m^3^	300 kg/m^3^	200–600 kg/m^3^
particle size	>400 µm	72 µm	<125 µm: min 90%
water solubility	Not applicable	400 g/L (25 °C)	>10 g/L (20 °C)

**Table 2:** Experimental plan.

Type of Admixture	Variation Levels of Polymer Additives			Additive Content Used by Weight from 100 w.p. of Emulsion		
	x_1_ (V)	x_2_ (H)	x_3_ (T)	X_1_ (Vinnapas 5034N)	X_2_ (Hostapur OSB)	X_3_ (Tylose MH60010 P4)
1	+	+	+	12.5	0.25	2
2	+	+	−	12.5	0.25	0.8
3	+	−	+	12.5	0.05	2
4	−	+	+	3.5	0.25	2
5	−	−	−	3.5	0.05	0.8
6	−	−	+	3.5	0.05	2
7	−	+	−	3.5	0.25	0.8
8	+	−	−	12.5	0.05	0.8
9	+	0	0	12.5	0.15	1.25
10	−	0	0	3.5	0.15	1.25
11	0	+	0	8	0.25	1.25
12	0	−	0	8	0.05	1.25
13	0	0	+	8	0.15	2
14	0	0	−	8	0.15	0.8
15	0	0	0	8	0.15	1.25

**Table 3:** Results of experiment.

No. of Experiment	Ostwald-de-Waele Function	Sna	Results of Experiment	
			η (Pa∙s) in γ′ = 1 s^−1^	|m|
1	4.94 − 0.59 γ′	0.052	142.6	0.59
2	2.69 − 0.64 γ′	0.077	12.9	0.64
3	4.21 − 0.52 γ′	0.064	67.36	0.52
4	4.24 − 0.53 γ′	0.092	73.04	0.53
5	2.03 − 0.58 γ′	0.091	6.71	0.58
6	3.73 − 0.55 γ′	0.053	42.14	0.55
7	2.55 − 0.68 γ′	0.118	10.69	0.68
8	3.39 − 0.72 γ′	0.113	25.2	0.72
9	3.64 − 0.53 γ′	0.023	37.7	0.53
10	3.33 − 0.55 γ′	0.021	27.25	0.55
11	3.25 − 0.64 γ′	0.039	23.94	0.64
12	3.11 − 0.56 γ′	0.025	21.89	0.56
13	4.56 − 0.61 γ′	0.043	94.89	0.61
14	2.42 − 0.65 γ′	0.038	10.56	0.65
15	3.61 − 0.58 γ′	0.017	36.08	0.58

**Table 4:** The relative increase in viscosity of emulsions.

Formula	Ostwald-de-Waele Function	η (Pa∙s) in γ′ = 1 s^−1^	Formula Number
P{VHT}/P{E}	2.91 − 0.01 lnγ′	18.4	(3a)
P{V}/P{E}	1.36 − 0.14 lnγ′	3.9	(3b)
P{H}/P{E}	0.52 − 0.11 lnγ′	1.7	(3c)
P{T}/P{E}	1.7 − 0.03 lnγ′	5.5	(3d)
(3b) + (3c) + (3d)	3.58 − 0.21 lnγ′	35.9	(3e)

## Data Availability

Data sharing not applicable.
